# Association Between Diabetes and Vertical Bone Defects in Periodontitis Using Cone Beam Computed Tomography: A Cross-Sectional Study in the Eastern Province, Saudi Arabia

**DOI:** 10.3390/clinpract15050095

**Published:** 2025-05-19

**Authors:** Eman Aljoghaiman, Abdullah Alzahrani, Rakan Albarqi, Saad Alqbbani, Hamad Alshiddi, Mishali AlSharief, Mohammed Alsaati, Faisal E. Al Jofi

**Affiliations:** 1 Department of Preventive Dental Sciences, College of Dentistry, Imam Abdulrahman bin Faisal University, P.O. Box 1982, Dammam 31441, Saudi Arabia; msalsharief@iau.edu.sa (M.A.); fealjofi@iau.edu.sa (F.E.A.J.); 2 College of Dentistry, Imam Abdulrahman bin Faisal University, Dammam 34212, Saudi Arabia; dr.abdullah.musharraf@gmail.com (A.A.); rakanalbarqi@hotmail.com (R.A.); saad503911@gmail.com (S.A.); 3 King Fahad University Hospital, Al Khobar 34445, Saudi Arabia; malsaati@iau.edu.sa

**Keywords:** periodontitis, diabetes, vertical bone defects, intrabony defects, cone beam computed tomography, cross-sectional study

## Abstract

**Background**: The association between diabetes and periodontal disease is well established, but its impact on intrabony periodontal defects remains unclear. Aims: This study examines the relationship between diabetes and intrabony periodontal defects using cone beam computed tomography (CBCT). **Methods:** A retrospective analysis of 99 CBCT images from Imam Abdurrahman bin Faisal University (2010–2022) was conducted. Intrabony periodontal defects were assessed, and logistic regression was used to analyze their association with diabetes. **Results**: Intrabony periodontal defects were detected in 66% of the sample, with 36% exhibiting multiple defects. Crude logistic regression showed a significant association between diabetes and intrabony periodontal defects (OR 3.986, 95% CI 1.454–10.922) and defect count (OR 3.382, 95% CI 1.430–8.003). However, multiple regression analysis did not confirm diabetes as an independent predictor (adjusted OR 0.740, 95% CI 0.087–6.314). **Conclusions:** Diabetes was not significantly associated with the prevalence or number of intrabony periodontal defects after adjusting for the confounders. However, these findings highlight the multifactorial nature of periodontal bone loss and the need for comprehensive patient assessment. Clinically, this underscores the importance of integrating both systemic and local periodontal evaluations in diabetic patients to improve early detection and personalized treatment planning. Further research with larger, more diverse samples and integrated clinical assessments is needed to refine our understanding of this relationship and enhance patient management.

## 1. Introduction

Oral diseases, such as periodontitis, and systemic conditions, including diabetes mellitus, obesity, and cardiovascular disease, share common multifactorial origins and behavioral risk factors [1]. The bidirectional relationship between oral and systemic health has been well established, particularly in the case of diabetes and periodontal disease [2].

Diabetes mellitus is a chronic metabolic disorder characterized by hyperglycemia due to insulin resistance, insufficient insulin secretion, or both [3]. The global prevalence of diabetes continues to rise, with an estimated 592 million cases projected by 2035 [4,5]. Diabetes is classified into several categories, including type 1, type 2, gestational diabetes, and other specific forms caused by genetic or pancreatic disorders [6]. Among these, type 2 diabetes accounts for 85–90% of cases, making it the most prevalent form [7,8]. Diagnosis relies on hemoglobin A1C (HbA1c), fasting plasma glucose (FPG), or the 2 h plasma glucose level after an oral glucose tolerance test (OGTT) [7,8]. Poor glycemic control increases the risk of complications, including neuropathy, nephropathy, cardiovascular disease, and oral health disorders [9,10].

Periodontitis is a chronic inflammatory disease that affects the supporting structures of the teeth, leading to gingival inflammation, attachment loss, alveolar bone destruction, and eventual tooth loss [11,12]. In Saudi Arabia, approximately 50% of adults experience some form of periodontal disease [13]. Major risk factors include poor oral hygiene, genetic predisposition, smoking, systemic diseases such as diabetes, and immune dysregulation [11,12,13]. Notably, diabetes is a major risk factor for periodontitis, increasing susceptibility to bacterial infections, exaggerating inflammatory responses, and delaying wound healing [14,15,16]. Evidence suggests that diabetic individuals have a threefold higher risk of developing periodontitis compared to that for non-diabetic individuals [17,18,19,20,21], with periodontitis recognized as the sixth most likely complication of diabetes [22].

The underlying mechanisms linking diabetes and periodontal disease include altered immune function, increased oxidative stress, and the accumulation of advanced glycation end-products (AGEs), all of which contribute to excessive tissue destruction and impaired bone remodeling [23,24,25,26,27]. Hyperglycemia disrupts the host immune response, affecting neutrophil chemotaxis, macrophage function, and osteoblast activity, thereby increasing susceptibility to periodontal breakdown and bone resorption [28,29].

Assessing periodontal bone loss is essential for diagnosing and managing periodontitis, particularly in diabetic patients. Traditional radiographic techniques, such as panoramic radiography (OPG) and full-mouth intraoral X-rays, provide valuable diagnostic information but are limited by their two-dimensional imaging, leading to distortion and inaccuracies in detecting bone loss [30,31,32]. Cone beam computed tomography (CBCT) addresses these limitations by offering high-resolution, three-dimensional imaging, enabling precise detection and quantification of intrabony defects [33,34,35,36].

Despite the well-established link between diabetes and periodontal disease, limited research has utilized CBCT to assess the prevalence and severity of vertical bone defects in diabetic individuals. While previous studies have evaluated the diagnostic accuracy of CBCT, few have specifically examined its role in comparing bone loss in diabetic vs. non-diabetic populations. This study aims to fill this gap by utilizing CBCT imaging to assess intrabony periodontal defects among diabetic and non-diabetic individuals, contributing to a better understanding of how diabetes influences periodontal bone loss.

## 2. Materials and Methods

### 2.1. Sample Selection and Study Design

The initiation of this project was contingent upon securing approval from the Institutional Review Board of Imam Abdulrahman bin Faisal University. CBCT images were retrieved systematically from the Imam Abdulrahman bin Faisal University’s CBCT library spanning the period from 2010 to 2022, all CBCT images were obtained utilizing the Cone Beam Computed Tomography machine (Carestream CS 9300, v3.7.1 Carestream Health Inc., Kodak, Rochester, NY, USA) forming a sample inclusive of individuals with periodontal involvement, both with and without diabetes. Inclusion was based on the availability of complete imaging data, clinical records, and demographic details. The sample size was determined based on the statistical power required to detect differences in bony defect prevalence between diabetic and non-diabetic groups. A standardized random sampling approach was employed to ensure representative data from the population.

This cross-sectional study targeted adults aged 20 to 80 years, with a specified sample size of 99 individuals. The broad age range was chosen to capture the variability in periodontal disease progression and its association with diabetes across different life stages. Since both conditions exhibit age-related changes in severity, including a wide range ensures a more representative assessment of how diabetes influences periodontal bone loss from early adulthood to older age.

### 2.2. Inclusion and Exclusion Criteria

For the Study Group: Dentate individuals with a minimum of two adjacent teeth, a diabetes diagnosis, presence of periodontitis, and availability of CBCT image files at Imam Abdurrahman bin Faisal University comprise the inclusion criteria. Exclusion criteria include subjects with evident pathology (such as a cyst or tumor), edentulous patients, individuals with genetic disorders or systemic diseases impacting bone condition, skeletal dysplasia, severe skeletal discrepancies, and those receiving medication affecting bone condition.

For the Control Group: The control group, matched in age, gender, and CBCT imaging to the study group, includes individuals without diabetes, with periodontitis, a minimum of two adjacent teeth, and availability of CBCT image files at Imam Abdurrahman bin Faisal University. The exclusion criteria for the control group align with those established for the study group.

### 2.3. Bony Defect Measurement Method

All CBCT images underwent examination using CS 3D imaging software v3.7.1 before being transferred to the three-dimensional software system (OSIRIX MD SOFTWARE CBCT) for volumetric measurement and analysis. A trained investigator was responsible for both the identification and measurement of the defects. The investigator performed volumetric measurements for each bony defect at comparable sites (mesial and distal; buccal and lingual) using OSIRIX software v12.0. This enabled the calculation of the total percentage of lost bony volume in relation to the adjacent bony height.

To qualify as a vertical bony defect, a defect must exceed 2 mm in its vertical distance from the cementoenamel junction (CEJ). This criterion aims to exclude the counting of normal alveolar bone level, typically situated 1.5 to 2.0 mm below the CEJ, as a bony defect.

Confirmation of the defect in CS 3D imaging software, viewed in three sequential views in both coronal and axial sections, preceded the measurement of the surface area of the bony defect (lost vertical bone) up to one decimal point using tools included in OSIRIX MD SOFTWARE CBCT.

Data collection, including the number of bony defects and the amount of bony loss within each defect, was conducted by the investigators. Two investigators performed data entry, with a third investigator ensuring the accuracy of the transferred data printed in the Excel sheet. Subsequently, the Excel sheet data was converted into an SAS file for analysis using SAS software, version 9.4.

### 2.4. Statistical Analysis

Statistical analyses was conducted using the Statistical Package SAS software, version 9.4 (SAS Institute Inc., Cary, NC, USA), with a significance level set at 0.05. Intra-examiner reliability was assessed by calculating the interclass correlation coefficient (ICC) for 10% of the randomly selected samples, measured one week apart. Investigators underwent a rigorous two-week calibration process to standardize defect identification and measurements. This included training sessions for using CS 3D imaging software and OSIRIX MD software for volumetric analysis. Ten randomly selected CBCT images were re-evaluated after one week to assess intra-examiner reliability. The interclass correlation coefficient (ICC) for these evaluations was 0.92, indicating high consistency.

The distribution of overall demographic factors and the prevalence of bony defects was reported, stratified by diabetic status. Simple logistic regression was applied to assess the unadjusted associations between diabetes and vertical bone defects. Multiple logistic regression was used to adjust for confounders, including age, gender, smoking, systemic diseases, and local factors. Adjusted odds ratios (ORs) with 95% confidence intervals (CIs) were reported to provide a comprehensive analysis of predictors.

## 3. Results

The demographic characteristics of the study population are summarized in Table 1. Among the 99 participants, the majority were female (53.5%), with the highest proportion falling within the 45–64 age range (51.5%). The prevalence of diabetes among the sample was 62.6%, and 69.7% of participants were diagnosed with periodontal disease. Additionally, 66% of participants exhibited intrabony periodontal defects, while 36% displayed multiple defects (≥2). These findings suggest that the study population primarily consisted of middle-aged individuals, with a significant proportion affected by diabetes and periodontal conditions.

To evaluate whether demographic and clinical factors were associated with the presence of intrabony periodontal defects, a Chi-squared test of independence was performed. The results, presented in Table 2, indicate a significant association between diabetes and the presence of intrabony periodontal defects (*p* = 0.0053). Other factors, including systemic disease, medication use, periodontal disease, and local factors, were also significantly associated with the presence of intrabony defects (*p* < 0.05). However, age, gender, and smoking status did not show a statistically significant association with intrabony periodontal defects. These findings suggest that systemic conditions, particularly diabetes and medication use, may play a more substantial role in the development of bone defects than traditional demographic risk factors

Further analysis was conducted to assess the association between these factors and the severity of intrabony periodontal defects, defined as having multiple lesions (≥2). The results, summarized in Table 3, show that diabetes, along with medication use and periodontal disease, was significantly associated with the presence of multiple intrabony periodontal defects (*p* = 0.0048). However, age, gender, systemic disease, and smoking status did not significantly influence the number of intrabony defects. These findings suggest that diabetes may contribute to both the presence and severity of intrabony periodontal defects, independent of other demographic factors.

To further evaluate the relationship between diabetes and intrabony periodontal defects, logistic regression analysis was performed to adjust for potential confounding factors. The crude logistic regression model revealed that diabetic participants exhibited 3.99 times higher odds of presenting with intrabony periodontal defects compared to non-diabetic participants (OR 3.986, 95% CI 1.454–10.922, *p* = 0.007). Similarly, the odds of having multiple intrabony periodontal defects (≥2 lesions) were significantly higher in diabetic individuals (OR 3.382, 95% CI 1.430–8.003, *p* = 0.005). However, after adjusting for confounders such as age, smoking, systemic disease, medication use, and local factors, multiple regression analysis did not confirm diabetes as an independent predictor of the presence (adjusted OR 0.740, 95% CI 0.087–6.314, *p* = 0.09) or the number (adjusted OR 1.454, 95% CI 0.139–15.169, *p* = 0.11) of defects, as shown in Table 4. While the crude analysis indicated a significant association, these results should be interpreted with caution due to the cross-sectional nature of the study. Further longitudinal studies are needed to confirm the role of diabetes in periodontal bone loss

## 4. Discussion

This study explores the correlation between diabetes and vertical periodontal defects using cone beam computed tomography (CBCT), providing critical insights into periodontal health management in diabetic patients. Among the study participants, 46.97% of diabetics exhibited vertical periodontal defects. The unadjusted odds ratio (OR) showed that diabetics were 3 to 4 times more likely to exhibit vertical periodontal defects than non-diabetics, a statistically significant finding. However, after adjusting for confounders, the OR suggested a 0.74 times higher likelihood, which was not statistically significant. Similarly, when assessing the number of defects, diabetics displayed a significantly higher risk of having more than two vertical periodontal defects, although the adjusted OR (1.5 times) did not reach statistical significance.

These findings align with those of prior studies, including those by Persson et al., which reported a higher prevalence of vertical periodontal defects in insulin-dependent diabetics compared to non-insulin-dependent diabetics [26]. Additionally, Ainamo et al. observed rapid bone loss in diabetic patients despite periodontal treatment, underscoring the challenges in managing periodontal health in this population [37]. Clinical attachment loss (CAL) and probing depth (PD) are well-documented indicators of periodontal disease severity and have been consistently reported as higher in diabetics compared to non-diabetics [38,39,40,41,42].

In our study, crude logistic regression revealed a significant association between diabetes and vertical bone defects. However, when adjusted for potential confounders, the association was no longer statistically significant in regards to the prevalence or number of defects between diabetic and non-diabetic groups. This underscores the multifactorial nature of periodontal disease and vertical periodontal defects. Correlating a singular factor, such as diabetes, with vertical periodontal defects proves challenging due to the complex interplay of various risk factors, including smoking, systemic influences, and local factors. A comprehensive understanding of these variables is essential for interpreting the prevalence of vertical periodontal defects in diabetic individuals. Also, the association found in the crude analysis indicates that diabetes may play a role in the development of vertical periodontal defects, but further longitudinal studies with larger sample sizes are needed to confirm this relationship and better understand the mechanisms behind bone loss in diabetic individuals

However, this study exhibits certain limitations that should be acknowledged. The reliance on CBCT imaging as the sole diagnostic method presents a limitation, as CBCT does not capture essential soft tissue parameters such as PD and CAL. While CBCT provides an accurate three-dimensional visualization of bony structures, integrating clinical periodontal assessments would improve the diagnostic accuracy of identifying vertical periodontal defects.

Another limitation is the lack of HbA1c measurements or other glycemic markers, preventing an evaluation of how diabetes control influences bone defects. Given the well-established impact of glycemic variability on bone metabolism and periodontal outcomes, future research should incorporate HbA1c levels to refine the understanding of diabetes severity and periodontal health.

Additionally, the assessment of local factors was limited. While food impaction and occlusal trauma were considered in the analysis, they were not clinically evaluated, which may have influenced the interpretation of vertical periodontal defects. Expanding data collection to include direct clinical examinations would provide a more comprehensive assessment of these factors.

The cross-sectional nature of the study and the sample size (99 CBCT images) also present constraints. While the sample was randomly selected to enhance representativeness, the limited sample size may reduce statistical power, particularly in multivariable regression models where multiple confounders are adjusted for. Although adjustment for key confounders was necessary to reduce potential bias and improve the validity of the findings, it may also have reduced the power of the model to detect statistically significant associations. Future research should employ larger, multi-center studies to validate these findings, ensure adequate power, and support more robust multivariable analyses.

Furthermore, the demographic composition, with a predominantly female sample (ages 45–46), may limit generalizability. Including a more diverse population with balanced age and gender representation would improve the external validity.

Despite these limitations, this study provides valuable insights into the association between diabetes and vertical periodontal defects, emphasizing the role of CBCT as a diagnostic tool. Future research should incorporate clinical periodontal parameters, such as probing depth, clinical attachment loss, and bleeding upon probing, alongside high-resolution radiographic assessments to enhance the accuracy of periodontal defect evaluation. Integrating CBCT with clinical examinations will provide a more comprehensive understanding of the impact of glycemic control, systemic health, and genetic predisposition on periodontal outcomes. These combined approaches will improve early detection and management strategies for periodontal disease in high-risk diabetic populations.

## 5. Conclusions

Despite the study’s limitations, it is reasonable to conclude that there is an increased likelihood of vertical defects in individuals with diabetes compared to those without diabetes. However, when accounting for other influencing factors, the observed correlation appears to be weak. Additional research is essential to provide a more nuanced understanding and a clearer definition of the relationship between vertical defects and diabetes. 

## Figures and Tables

**Table 1:** Demographic characteristics of study participants.

	Total (n)	Percentage %
**Age (N = 99)**		
18–44	39	39.39
45–64	51	51.51
65+	9	9.09
**Gender (N = 99)**		
Male	46	46.46
Female	53	53.53
**Education (N = 99)**		
Illiterate	2	2.02
Primary school	6	6.06
Middle school	5	5.05
High school	49	49.49
Diploma	3	3.03
Bachelor’s	33	33.33
PhD	1	1.01
**Marital Status (N = 99)**		
Single	19	19.19
Married	80	80.80
**Diabetic (N = 99)**		
No	37	37.37
Yes	62	62.62
**Systemic disease (N = 99)**		
No	91	91.91
Yes	8	8.08
**Medications (N = 99)**		
No	63	63.63
Yes (diabetes medications)	36	36.36
**Smoker (N = 99)**		
No	76	76.76
Yes	23	23.23
**Periodontal disease (N = 99)**		
No	30	30.30
Yes	69	69.69
**Vertical bone defect (N = 99)**		
No	33	33.33
Yes	66	66.66
**Local factor (N = 99)**		
No	77	77.77
Yes	22	22.22
**Vertical bone defect number (N = 99)**		
<2	63	63.63
≥2	36	36.36

**Table 2:** Demographic predictors of presence of intrabony periodontal defects.

	Total (n)	Without Vertical Bone Defect	With Vertical Bone Defect	*p* Value *
Age (N = 99)				
18–44	39	39.39	39.39	0.7452
45–64	51	54.55	50.00	
65+	9	6.06	10.61	
Gender (N = 99)				
Male	46	51.52	43.94	0.4762
Female	53	48.48	56.06	
Education (N = 99)				
Illiterate	2	3.03	1.52	0.9773
Primary school	6	6.06	6.06	
Middle school	5	3.03	6.06	
High school	49	51.52	48.48	
Diploma	3	3.03	3.03	
Bachelor’s	33	33.33	33.33	
PhD	1	0	1.52	
Diabetic (N = 99)				
No	62	81.82	53.03	0.0053
Yes	37	18.18	46.97	
Systemic disease (N = 99)				
No	91	100	87.88	0.0370
Yes	8	0	12.12	
Medications (N = 99)				
No	63	84.85	53.03	0.0019
Yes (diabetes medications)	36	15.15	46.97	
Smoker (N = 99)				
No	76	81.82	74.24	0.4001
Yes	23	18.18	25.76	
Periodontal disease (N = 99)				
No	30	51.52	19.70	0.0012
Yes	69	48.48	80.30	
Local factor (N = 99)				
No	77	93.94	69.70	0.0062
Yes	22	6.06	30.30	

* Rao–Scott Chi-squared test.

**Table 3:** Demographic predictors of vertical bone defects ≥ 2 among study participants.

	Total (n)	Vertical Bone Defect Number < 2	Vertical Bone Defect Number > 2	*p* Value *
**Age (N = 99)**				
18–44	39	36.51	44.44	0.5573
45–64	51	52.38	50.00	
65+	9	11.11	5.56	
**Gender (N = 99)**				
Male	46	47.62	44.44	0.7606
Female	53	52.38	55.56	
**Education (N = 99)**				
Illiterate	2	3.17	0	0.6688
Primary school	6	6.35	5.56	
Middle School	5	6.35	2.78	
High school	49	46.03	55.56	
Diploma	3	1.59	5.56	
Bachelor’s	33	34.92	30.56	
PhD	1	1.59	0	
**Diabetic (N = 99)**				
No	62	73.02	44.44	0.0047
Yes	37	26.98	55.56	
**Systemic disease (N = 99)**				
No	91	90.48	94.44	0.4859
Yes	8	9.52	5.56	
**Medications (N = 99)**				
No	63	76.19	23.81	0.0006
Yes (diabetes medications)	36	23.81	58.33	
**Smoker (N = 99)**				
No	76	76.19	77.78	0.8572
Yes	23	23.81	22.22	
**Periodontal disease (N = 99)**				
No	30	46.03	2.78	<0.0001
Yes	69	53.97	97.22	
**Local factor (N = 99)**				
No	77	84.13	66.67	0.0444
Yes	22	15.87	33.33	

* Rao–Scott Chi-squared test.

**Table 4:** Logistic regressing analysis for the relationship between diabetes, vertical bony defects, and number of vertical bony defects.

Outcome	Main Predictor	Crude Model	*p*-Value	Adjusted Model	*p*-Value
		Odds Ratio (95% Confidence Interval)		Odds Ratio (95% Confidence Interval)	
Vertical bone defect Yes vs. No	**Diabetic**	3.986 (1.454–10.922) *	0.0072	0.740 (0.087–6.314)	0.7831
Vertical bone defect number <2, vs. ≥2		3.382 (1.430–8.003) *	0.0055	1.454 (0.139–15.169)	0.7542

* Asterisk indicates statistically significant at *p* < 0.05 level.

## Data Availability

The data presented in this study are available on request from the corresponding author(s) but are not publicly accessible due to ethical and confidentiality restrictions.
